# Neuroimmune Regulation in Sepsis-Associated Encephalopathy: The Interaction Between the Brain and Peripheral Immunity

**DOI:** 10.3389/fneur.2022.892480

**Published:** 2022-06-27

**Authors:** Yu-xiao Liu, Yang Yu, Jing-peng Liu, Wen-jia Liu, Yang Cao, Run-min Yan, Yong-ming Yao

**Affiliations:** ^1^Translational Medicine Research Center, Medical Innovation Research Division and Fourth Medical Center of the Chinese PLA General Hospital, Beijing, China; ^2^Department of Neurosurgery, The Chinese PLA General Hospital, Beijing, China; ^3^Department of Traditional Chinese Medical Science, Sixth Medical Center of the Chinese PLA General Hospital, Beijing, China; ^4^State Key Laboratory of Proteomics, Beijing Proteome Research Center, National Center for Protein Sciences, Beijing Institute of Lifeomics, Beijing, China

**Keywords:** sepsis-associated encephalopathy, neuroendocrine–immune network, hypothalamic–pituitary–adrenal axis, cholinergic anti-inflammatory pathway, neuroinflammation

## Abstract

Sepsis-associated encephalopathy (SAE), the most popular cause of coma in the intensive care unit (ICU), is the diffuse cerebral damage caused by the septic challenge. SAE is closely related to high mortality and extended cognitive impairment in patients in septic shock. At present, many studies have demonstrated that SAE might be mainly associated with blood–brain barrier damage, abnormal neurotransmitter secretion, oxidative stress, and neuroimmune dysfunction. Nevertheless, the precise mechanism which initiates SAE and contributes to the long-term cognitive impairment remains largely unknown. Recently, a growing body of evidence has indicated that there is close crosstalk between SAE and peripheral immunity. The excessive migration of peripheral immune cells to the brain, the activation of glia, and resulting dysfunction of the central immune system are the main causes of septic nerve damage. This study reviews the update on the pathogenesis of septic encephalopathy, focusing on the over-activation of immune cells in the central nervous system (CNS) and the “neurocentral–endocrine–immune” networks in the development of SAE, aiming to further understand the potential mechanism of SAE and provide new targets for diagnosis and management of septic complications.

## Introduction

Sepsis is a common complication in patients with burns or wound injury, infection, shock, and major surgery. It is a dysregulated host response induced by severe infection, which may develop into multiple organ failure and eventually lead to death. In 2020, Rudd et al. (1) analyzed the onset and mortality of sepsis from 1997 to 2017 and found that there were about 48.9 million patients in septic shock in the world. Now, sepsis is regarded as a prominent unsolved problem around the world due to its high incidence, fatality rate, and medical cost.

The multiple organ dysfunction induced by sepsis results in an imbalance of the immune response, which may aggravate the clinical symptoms of sepsis. As the primary organ affected by inflammation in sepsis, the brain is not only critically involved in immune regulatory response but also vulnerable to injury, which may lead to sepsis-associated encephalopathy (SAE). Of note, SAE is a diffuse brain dysfunction secondary to sepsis, with an incidence of more than 70% in patients in septic shock, especially in the elderly people, neonates, and patients with a chronic illness (2). SAE can lead to long-term neurological damage, such as anxiety, memory impairment, and consciousness disorders. It was reported that the mortality rate of patients with septic encephalopathy appeared to be much higher than that of patients without encephalopathy (3). However, the diagnostic criteria of septic encephalopathy have not been well established until now due to the clinical use of sedatives and the existence of a potential neurological disorder. Therefore, it is of great significance to further investigate the pathogenesis of SAE and recognize early the development of SAE.

With marked advancements in neuroscience, the potential role of the neuroendocrine–immune network in the immune response of sepsis has been gradually revealed. It is documented that circulating inflammatory mediators and hormones cause dysfunction of the central nervous system (CNS) in sepsis by inducing the activation of glia and the death of neural cells. In turn, the CNS reacts to the peripheral immune system through the neuroendocrine–immune network, which forms a feedback regulatory loop in the central–peripheral immune system. Then, the CNS triggers a neural reflex and regulates neurotransmitters, neurohormones, and cytokines produced by synapses. Meanwhile, the nervous system extends the nerve fibers to the peripheral organs, which may regulate the secretion function of internal or external secretory glands and maintain the homeostasis of the environment. The central–peripheral immune system regulates the inflammatory response mainly by the hypothalamic–pituitary–adrenal (HPA) axis and the cholinergic anti-inflammatory pathway (CAP), which cooperate to maintain a moderate immune state according to the needs of the body (4). For example, α7 nicotinic acetylcholine receptors (α7 nAChRs), the important component of the CAP, play key roles in central–peripheral immune regulation in the setting of sepsis. The activation of α7 nAChRs inhibits the central and peripheral inflammatory responses and prevents immunosuppression by affecting the differentiation of microglia/macrophages and decreasing the production of inflammatory cytokines. These processes are involved in several signaling pathways, including Janus kinase (JAK)2/signal transducer and activator of transcription (STAT)3 signaling and toll-like receptor 4 (TLR4)/nuclear factor kappa-B (NF-κB) signaling (5–8). However, the disruption of the neuroendocrine–immune network results in an aberrant immune response and aggravates the dysfunction of sepsis. For example, Boomer et al. (9) reported that SAE caused aggressive immunosuppression through the HPA axis, which might induce multiple organ failure and give rise to a vicious circle of immune dysfunction. Souza pointed out that the downregulation of α7 nAChRs in the hypothalamus induced by lipopolysaccharide (LPS) aggravated the neuroinflammatory and metabolic disorders in the brain (10). As an important regulator of the neuroendocrine–immune network, the CNS might be a useful target for the management of septic complications.

Here, we summarize the main physiological alterations of the brain in SAE and discuss the underlying mechanisms that might accelerate the sepsis-induced brain damage, especially focusing on the function of the neuroendocrine–immune network in the pathogenesis of SAE.

## Clinical Symptoms of SAE

SAE is regarded as a cognitive dysfunction induced by sepsis without obvious nervous system infection or structural injury. Due to the lack of a consensus definition of the SAE, the incidence of SAE varied widely among the studies. It has been reported that 8% to more than 70% of patients in septic shock in the ICU may have encephalopathy, only 19% of the patients in the ICU have a normal electroencephalogram (EEG) with alpha rhythm, and 80% of them show epileptic or abnormal EEG (11). The symptoms of septic encephalopathy include memory decline, attention loss, orientation, irritability, and trance. To be specific, SAE may cause long-term neurological deficits, with 10–20% of patients in septic shock showing cognitive deficits, 10–30% of them showing anxiety and stress disorders, and 31–70% of patients having chronic pain. In addition, SAE may result in epileptic seizures, delirium, mild or deep unconsciousness, and even coma (11–13).

The severity of SAE ranges from temporary to permanent brain dysfunction. According to the recent observations of the symptoms, SAE can be divided into acute, sub-acute, or chronic types. The acute SAE can be controlled with patient improvement (14). It is considered a sub-acute or chronic type when the symptoms persist for months or years, and the psychological and cognitive deficits induced by SAE greatly affect the life quality of patients for a long time. Previous studies indicated that mild neurological symptoms in 20–40% of patients with SAE, such as memory changes, depression, anxiety, or cognitive disorders, might persist for 1 year (15, 16). Even worse, the long-term neurological abnormalities not only increase sepsis-induced fatal outcomes but also induce suicidal behaviors within 2 years of recovery (17, 18). Moreover, SAE has an impact on the link between the brain parenchymal and peripheral circulations, which may decrease the blood vessel and capillary densities and cause microcirculation abnormalities (19).

## The Immune Response of CNS in the Development of SAE

**Figure 1:**
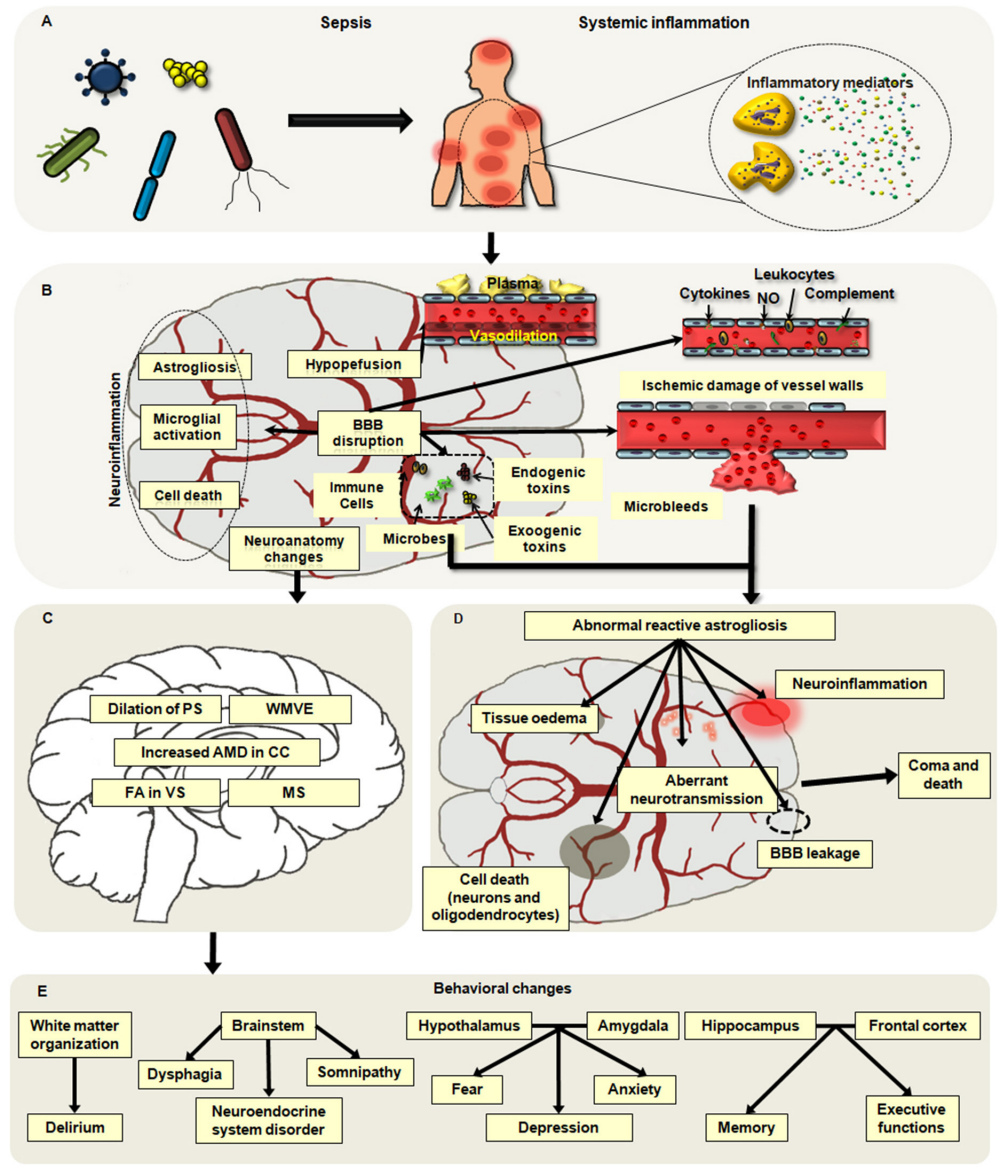
Main pathophysiological alterations in sepsis-associated encephalopathy (SAE). **(A,B)** Neuroinflammation, hypoperfusion, neuroanatomy changes, and neuronal death are major causes of SAE. Neuroinflammation mainly includes the infiltration of neutrophils and the activation of microglia and astrocytes, which may aggravate brain excitotoxicity and blood–brain barrier (BBB) disruption. Hypoperfusion is frequently observed in patients with SAE, and it can be induced by abnormal vasodilation, impaired autoregulation of the cerebral blood flow (CBF), and inflammatory insults. Apoptosis and pyroptosis are common ways of neuronal cell death associated with brain dysfunction in sepsis. **(C)** The changes in neuroanatomy including the white matter vasogenic edema (WMVE), myelin separation (MS), dilation of the perivascular spaces (PC), increased axial water diffusion (AWD) in the corpus callosum (CC), and fractional anisotropy (FA) in the ventral striatum (VS) are noticed in patients with SAE. **(D)** The abnormal astroglial reactivity is regarded as a key contributor to the development of SEA. In the setting of sepsis, the BBB is disrupted by severe systemic inflammation, which may result in ischemic damage of vessel walls, microabscess formation, and infiltration of microbes, immune cells, and toxins into the brain parenchyma. These alterations can cause the abnormal reactivation of astrocytes, which will deteriorate brain injuries including BBB breakdown, neuroinflammation, tissue edema, cell death, and aberrant neuro-transmission. **(E)** Disorder in various regions of the brain may lead to different behavior alterations secondary to septic challenges.

It is believed that the pathophysiological factors of cerebral dysfunction secondary to sepsis mainly include neuroinflammation, blood–brain barrier (BBB) impairment, disorders of brain perfusion, alterations in neurotransmitters, and changes in neuroanatomy and cell death (20, 21). Among them, four types of factors are especially involved with brain dysfunction: the aggressive inflammation of the brain, ischemic injury, the changes in neuroanatomy, and the death of neural cells (Figures 1A,B).

Notably, the disorders of immune responses in the CNS are the major cause of SAE, which play key roles in the dysregulated peripheral immunity and multiple organ dysfunction syndromes (MODS) following a septic challenge. Ischemic injury, the change in the microstructure in the brain, and the neuron death contribute to the brain damage induced by the uncontrolled inflammation and the difficulty of SAE treatment.

### Effect of Neuroinflammation on Immune Cells of the Brain

Uncontrolled neuroinflammation is a significant indicator of septic encephalopathy. During the process of sepsis, peripheral and local inflammation can induce the migration of neutrophils into the brain and activate the astrocytes and microglia (22–25). Then, the activated immune cells in the CNS may release the excessive proinflammatory mediators, alter oxidative and nitrosative stress, and increase neurotransmitters, which aggravate the central and peripheral inflammatory responses.

### Neutrophil Infiltration

The progress of leukocyte recruitment includes trapping, slow rolling, adhesion to endovascular crawling, and migrating. As the hallmark of inflammation, the infiltration of neutrophils is found in the CNS at the early stage of sepsis, while few of them are observed in the normal brain. The neutrophils are induced to damage tissue sites by a variety of inflammatory mediators, including interleukins (ILs), tumor necrosis factor (TNFs), angiotensin, and the complement cascade factors (26–29). In this process, neutrophils migrate and adhere to endothelial cells by selectins and integrins. Then, they activate the cell adhesion molecules on vascular endothelial cells, crawl on the vascular endothelium, and search for interendothelial junctions to migrate through the endothelial barrier. Thereafter, the neutrophils pass through the glia limitans and interact with glial cells at the parenchyma. The accumulation of neutrophils in the CNS may damage the cerebrovascular function and the neural cells by releasing oxygen-free radicals and increasing the volume of erythrocytes (30, 31).

Norman et al. (32) reported that neutrophils were significantly recruited by the brain during the acute inflammatory phase of sepsis, and the transmigration of neutrophils across the endothelial barrier was the main cause of vascular barrier breakdown. Recent studies documented that SB225002 (the antagonist of CXCR2) or kynurenic acid could protect the brain against neutrophil activation and BBB permeability changes in septic animals (33, 34). However, neutrophil recruitment was not involved in cognitive impairment (35). The activated microglia and astroglial cells may be responsible for the dysfunctions of the synaptic and intracerebral communication networks.

### Microglia

Microglia accounts for 6%-18% of human brain cells (36). As the resident immune cells in the brain, the microglia is the first cell to respond to neuroinflammation. They regulate the functions and inflammation of the brain by secreting cytokines and interacting with other neural cells.

In normal conditions, the microglia with branched morphology is resting, which is responsible for micro-environment surveillance. After activation by pathological insults, microglia reveals quick and prominent alterations in morphology, metabolism, and function. A series of studies suggested that microglia was consistently activated in animal models and patients of sepsis (11, 37). For example, the increasing microglia with amoeboid shape was observed in the hippocampus and cortex of the patients who died of septic shock (38, 39).

It has been documented that peripheral inflammation activates microglia with the help of the inflammatory mediators, the interaction between microglia and adjacent cells, and neurotransmitters (40). In the development of sepsis, cytokines produced by peripheral immune cells such as TNF-α and IL-1β transmit signals through the damaged BBB and activate microglia. For example, Ye et al. reported that during sepsis, IL-17A secreted by peripheral immune cells could activate microglia, and then the inflammatory mediators produced by activated microglia enhanced the release of IL-17A from immune cells, which created a vicious cycle to amplify the brain inflammation (41). Adjacent cells including leptomeningeal cells or vascular endothelial cells also augmented the activation of microglia, and the activated endothelial cell stimulated and guided microglia into the inflammatory brain during sepsis by the upregulation of CX3CL1 (42). Moreover, the neurotransmitters such as acetylcholine (Ach), nicotine, and glutamate regulated LPS-induced microglial activation (43).

A series of studies have indicated that the activation of microglia in sepsis results in brain dysfunctions including delirium, memory impairment, acute brain oxidative damage, and long-term cognitive impairment by secreting a large quantity of proinflammatory cytokines, upregulating the expression of multiple enzymes and neurotransmitters, and changing the brain energy metabolism in sepsis (41, 43, 44). For example, a mild level of glutamate is produced by neural cells to maintain the homeostatic state of the brain, while activated microglia may release toxic amounts of glutamate and induce long-term cognitive dysfunction after sepsis (45). Furthermore, microglia can significantly promote glucose uptake and glycolysis by expressing glucose transporters 1 (GLUT), increasing the production of IL-1β, and upregulating oxidative/nitrosative enzymes in inflammation (46).

The growing evidence has proved that the difference in the microenvironment may lead to the heterogeneity of microglia. There are two kinds of activated phenotypes of microglia named M1 and M2 subpopulations, respectively. The microglia with different phenotypes show either adverse or advantageous effects on neurological function (47). It is well known that M2 microglia can protect the neural cells from inflammatory insults, while the activated M1 microglia can aggravate neuroinflammation and oxidative stress by increasing inflammatory mediators and decreasing mitochondrial oxygen consumption and ATP production in sepsis. Thus, the increase in M2 microglia or the inhibition of M1 microglia may alleviate inflammation and brain dysfunction following septic challenge. Yan et al. (47) found that the induction of microglia from the M1 to the M2 phenotype by IL-4/IL-13 stimulation inhibited the release of inflammatory cytokines and alleviated the brain dysfunction in SAE by increasing the mitochondrial content. Thus, it could be a promising therapeutic method to improve the long-term cognitive impairment induced by sepsis by regulating the polarization of microglia and inhibiting the secretion of proinflammatory mediators.

### Astrocytes

Astrocytes are of great importance to maintain the homeostasis of the brain, which is associated with BBB permeability, neurotransmitter metabolism, synapse connectivity and plasticity, and brain fluid balance (48). Under physiological conditions, the morphology and function of astrocytes are region-dependent and heterogeneous. Astrocytes are essential components of the BBB and the glia-neurovascular barrier in the gray matter protoplasm, and they regulate the neurotransmitter catabolism by specific transporters and provide neurotransmitter precursors for neurons. Importantly, astrocytes are responsible for the formation of the endocranial secretory system, which releases neuromodulators, trophic factors, and hormones.

The pathological changes of astrocytes mainly include astrogliosis and astrodegeneration. It has been demonstrated that reactive astrogliosis is caused by brain injury, and the main alterations of activated astroglial cells are hypertrophic and upregulated expression of the glial fibrillary acidic protein (GFAP) and vimentin (48–50). The degree of astrogliosis relies on the severity of the damage, which is regulated by cellular signaling pathways such as the JAK/STAT and NF-κB pathways (51). The proliferation of astrocytes in the cerebral cortex is one of the common histopathologic changes in the brain secondary to septic insults. Several studies confirmed that reactive astrogliosis was observed in the brain tissues during neuroinflammation induced by LPS exposure, which was associated with synaptic deficits and depressive-like behaviors in mice (25, 52). The activation of astrocytes enhanced the release of inflammatory mediators and aggravated CNS inflammation, and it was regulated by expressions of p21, NF-κB, and inducible nitric oxide synthase (iNOS) in astrocytes from septic animals. Likely, astrocytes might take part in the formation of the brain microabscesses and the infiltration of inflammatory cells in SAE and CNS injury (25, 53). Astrocytes induced the migration of leukocytes into the damaged sites of the brain by upregulating the leukocyte adhesion molecules, destroying the integrity of tight junction (TJ), and promoting the formation of transendothelial cell channels (53, 54). Moreover, astrocytes are responsible for the collapse of the BBB. In the setting of sepsis, cytokines released by astrocytes could inhibit the expression of TJ proteins in endothelial cells and thus aggravate BBB breakdown (55). In LPS-induced animal models, the morphology, transcriptional profile, and phenotype of astrocytes were altered, including early reactivity, astrocytic end-feet remodeling, and even astrocytic loss, thereby resulting in the disruption of BBB (56–58). Being a crucial part of the central immune system, the abnormality of astrocytes accelerates the development of neuroinflammation and aggravates brain dysfunction (Figure 1D). Astrocytes determine the severity and level of brain damage, and astroglial reactivity is regarded as a defining factor in the pathophysiology of SAE (25). Nevertheless, the process of SAE in humans is more complicated than that in animal models, which is associated with age and pathological background. So, deeper exploration should be carried out to clarify the function of the astrocytic network in SAE.

### Ischemic Injury and Alteration in Neuroanatomy

Many studies have demonstrated that ischemic injury often occurs in the brain in the setting of sepsis (59–61). The clinical data have been shown that cerebral blood flow (CBF) is significantly reduced in patients with SAE (59). For instance, Sharshar et al. (60) analyzed the brain tissues of the patients dying from septic shock and found the incidence of cerebral ischemia in septic shock to be 100%. Szilárd et al. (62) reported that the CBF rate was slower in patients with septic encephalopathy than in normal controls by transcranial Doppler ultrasound. Increasing evidence proved that the intravenous injection of bacterial LPS to human volunteers markedly resulted in the reduced global CBF and the damaged cerebral autoregulation of the middle cerebral artery (MCA) blood velocity, in turn contributing to the sepsis-associated delirium and edema (61, 63, 64).

Strikingly, the results from the experimental models of sepsis are controversial. Several studies revealed the decreased blood perfusion distribution in animals after systemic administration of LPS, while others reported the increased CBF in cortical MCA territories after LPS challenge (61, 65). It is speculated that these alterations in CBF during sepsis are due to the impaired autoregulation of CBF, and the discrepancy in different studies might be associated with the model-building methods and the dose of drugs.

Due to the reversibility of SAE, most studies on histopathology of SAE did not observe the obvious changes in deeper structures and the spinal cord of the brain (66). Nevertheless, emerging evidence has documented that the disorder of cerebral autoregulation may contribute to edema and white matter change in sepsis (67, 68). Alterations in neuroanatomy have been noted in the patients with SAE including the white matter vasogenic edema, myelin separation, dilation of the perivascular spaces, incremental axial water diffusion in the corpus callosum, and fractional anisotropy in the ventral striatum (39, 68, 69). The dysfunction in various regions of the brain may cause different behavior disorders. The impairment of white matter organization causes delirium (70). The malfunction of the brainstem can lead to dysphagia, neuroendocrine system dysregulation, and somnipathy, which appear to be associated with multiple organ dysfunction and higher mortality in patients (71–73). The disorder of the hypothalamus or the amygdala is related to behavioral changes including depression, fear, and anxiety (74, 75). As is known, the hippocampus and the frontal cortex play vital roles in memory formation; thus, the disruption of the hippocampus and the frontal cortex causes the deterioration of memory and executive functions (76–78) (Figures 1C,E). Until now, the sepsis-induced alterations in the brain microstructure are largely unknown, but it is predictable that these changes may contribute to the development of brain dysfunction and long-term cognitive impairment. Therefore, further understanding of the neuroanatomical approach is of significance to explore the clinical features and psychocognitive disorders of SAE.

### Cell Death in SAE

Increasing studies have indicated that cell death is an important contributor to brain dysfunction during SAE. The apoptosis and pyroptosis of brain cells induced by inflammation are critically involved in the onset and progression of SAE.

Apoptosis is a sort of regulated cell death accompanied by contraction of the cells and foam cell formation in the cell membrane. It is well accepted that mitochondria are major apoptosis-associated organelles. In the development of sepsis, mitochondrial dysfunction is found in various regions of the brain, especially in the hippocampus, which may cause the imbalance of oxygen/nitrogen reactive species, hippocampus cell apoptosis, and severe neurocognitive impairment. Elevated neuronal apoptosis is characterized by enhanced pro-apoptotic proteins, which are released from mitochondrial cleaved caspase levels. Omi/HtrA2, a serine protease in mitochondria, regulates apoptosis of hippocampus cells by translocation from mitochondria into the cytoplasm in cecal ligation and puncture (CLP)-induced sepsis (79). More recently, the mitochondrial isomerase cyclophilin D (CypD) was found to be associated with mitochondrial dysfunction and cell apoptosis in SAE. The knockout of CypD could protect the brain cells from apoptosis by decreasing the opening mitochondrial permeability transition pore and inhibiting the free radicals from mitochondria, which were able to alleviate the brain damage and improve the survival of CLP-induced SAE (80). Similarly, the inhibition of ceramide significantly attenuated neuronal apoptosis and cognitive impairment of septic animals (81).

There is an obvious link between the apoptosis of neuronal cells and the abnormality of proinflammatory signaling pathways, including NF-κB, mitogen-activated protein kinase (MAPK), and brain-derived neurotrophic factor (BDNF)/tyrosine kinase (Trk)B during SAE. The activation of NF-κB is regarded as a central event in sepsis, which may trigger the inflammatory mediator networks. Some studies reported that the activation of NF-κB was related to increases in the mortality rate and poor clinical outcomes in sepsis. Accordingly, the inhibitors of NF-κB could attenuate LPS-induced long-term potentiation in the dentate gyrus and ischemia-induced neuronal apoptosis (82). Furthermore, p65, a subunit of the NF-κB heterodimer, can induce the transcription of microglia Nod-like receptor protein 3 (NLRP3) by directly binding to the promoter region of NLRP3 in SAE. As an important component of the NLRP3 inflammasome, the activation of NLRP3 induces cell apoptosis by regulating the expression of BCL and Bax. There is accumulating evidence demonstrating that downregulated activation of the NF-κB/p65-induced NLRP3 inflammasome inhibits the neuron apoptosis induced by LPS *in vivo* and *in vitro* (83–85).

MAPK plays a key role in inflammation-induced apoptosis. p38 MAPK is activated in the brain accompanied by the upregulated phosphorylation of MAPKAPK2 upon sepsis. Cell apoptosis in the cortex and the hippocampus of CLP animals could be alleviated by p38 MAPK inhibitors (86). Zhou et al. (87) found that the neuronal apoptosis and autophagy were highly related to the p38 MAPK signaling pathway in the CLP-induced sepsis model, while immunity-related GTPase M1 (IRGM1) partially ameliorated neuronal apoptosis by the p38 MAPK signaling pathway. BDNF is regarded as an important component to retain activity-dependent synaptic plasticity. It has been documented that the inactivation of BDNF/TrkB signaling following a brain injury is involved in apoptosis and autophagy of neuronal cells. For instance, the activation of BDNF/TrkB alleviated brain damage induced by SAE, while the abnormality of BDNF/TrkB signaling was associated with brain dysfunction in aging mice (88, 89). Likely, treatment with emodin, a chemical compound from rhubarb, obviously decreased the apoptosis of hippocampal neurons in CLP mice through the activation of the BDNF/TrkB pathway (90).

Pyroptosis is defined as regulated necrosis with rapid damage to the plasma membrane and the release of intracellular contents (85), and it is a common pattern of cell death in sepsis, which can aggravate inflammation. Many studies have confirmed the pyroptosis of peripheral immune cells in patients in septic shock (91, 92). Recently, we observed pyroptosis of splenic dendritic cells (DCs) in CLP-induced septic mice (93). Similarly, Xu et al. noticed the pyroptosis of neural cells in septic mice and found that the inhibition of pyroptosis could alleviate brain damage and cognitive impairment in SAE. They speculated that pyroptosis of neural cells was induced by inflammatory mediators, which were secreted by the peripheral blood into the CNS during SAE (94). The pyroptosis can be mediated through the canonical or non-canonical signaling pathway. In the non-canonical signaling pathway, LPS can directly activate caspase-11,−4, and−5 to cleave the critical pyroptosis executioner, gasdermin D (GSDMD), and form N-terminal GSDMD (GSDMD-NT). Then, GSDMD-NT becomes a ring-like structure and forms holes in the cell membrane, which causes the production of IL-1β and IL-18, and leads to cell lysis. The canonical signaling pathway plays a potential role in the pathogenesis of SAE, and the inhibition of pyroptosis may be a useful therapeutic target for SAE (95, 96).

The canonical signaling pathway relies on the inflammasome pre-activation. Then, the inflammasome can activate caspase-1 by the adaptor apoptosis speck-like protein, which cleaves GSDMD to form GSDMD-NT (97, 98). As the pivotal canonical inflammasome, the NLRP3 inflammasome is highly associated with brain damage during SAE. With this regard, expressions of NLRP3, GSDMD-NT, and cleaved caspase-1 were augmented in the brain of septic mice, and the upregulation of NLRP3, caspase-1, and gasdermin-D in the hippocampus resulted in the cognitive deficits in the mice model of SAE by inducing pyroptosis (99). Recently, we found that sestrin 2 improved the outcome of sepsis by decreasing the NLRP3/caspase-1-dependent pyroptosis, and it was closely related to the protein kinase RNA (PER)-like ER kinase (PERK)-activating transcription factor (ATF)4-C/EBP homologous protein (CHOP) pathway (95). Moreover, Lei et al. (100) explored the nexus between autophagy and pyroptosis in mice with SAE, and the results showed that both autophagy and pyroptosis were involved in the development of SAE in mice, and pannexin-1 decreased the occurrence of pyroptosis by autophagy. Collectively, it was suggested that both the canonical and non-canonical pathways of cell pyroptosis are associated with cell apoptosis induced by LPS (95). However, the exact functions of the canonical and non-canonical pyroptosis together with the networks among pyroptosis, apoptosis, and autophagy in SAE are required in further studies.

## Neuroimmune Regulation in SAE

Reciprocal interactions between the central and peripheral immune systems are regarded as crucial parts of the host's response to septic insults. When systemic inflammatory responses are induced by severe trauma or infection, the peripheral inflammatory signal is sent to the CNS. The CNS regulates immune system compensatory changes and maintains homeostasis after perception and integration of peripheral signals through sympathetic, parasympathetic, and HPA gland axial. The aberrant responses of neuroendocrine–immune networks are responsible for the immune disruption and the worse outcome during sepsis. It is well known that the neural central–endocrine–immune networks are mainly composed of HPA and CAP.

### Hypothalamic–Pituitary-Adrenal Axis

**Figure 2:**
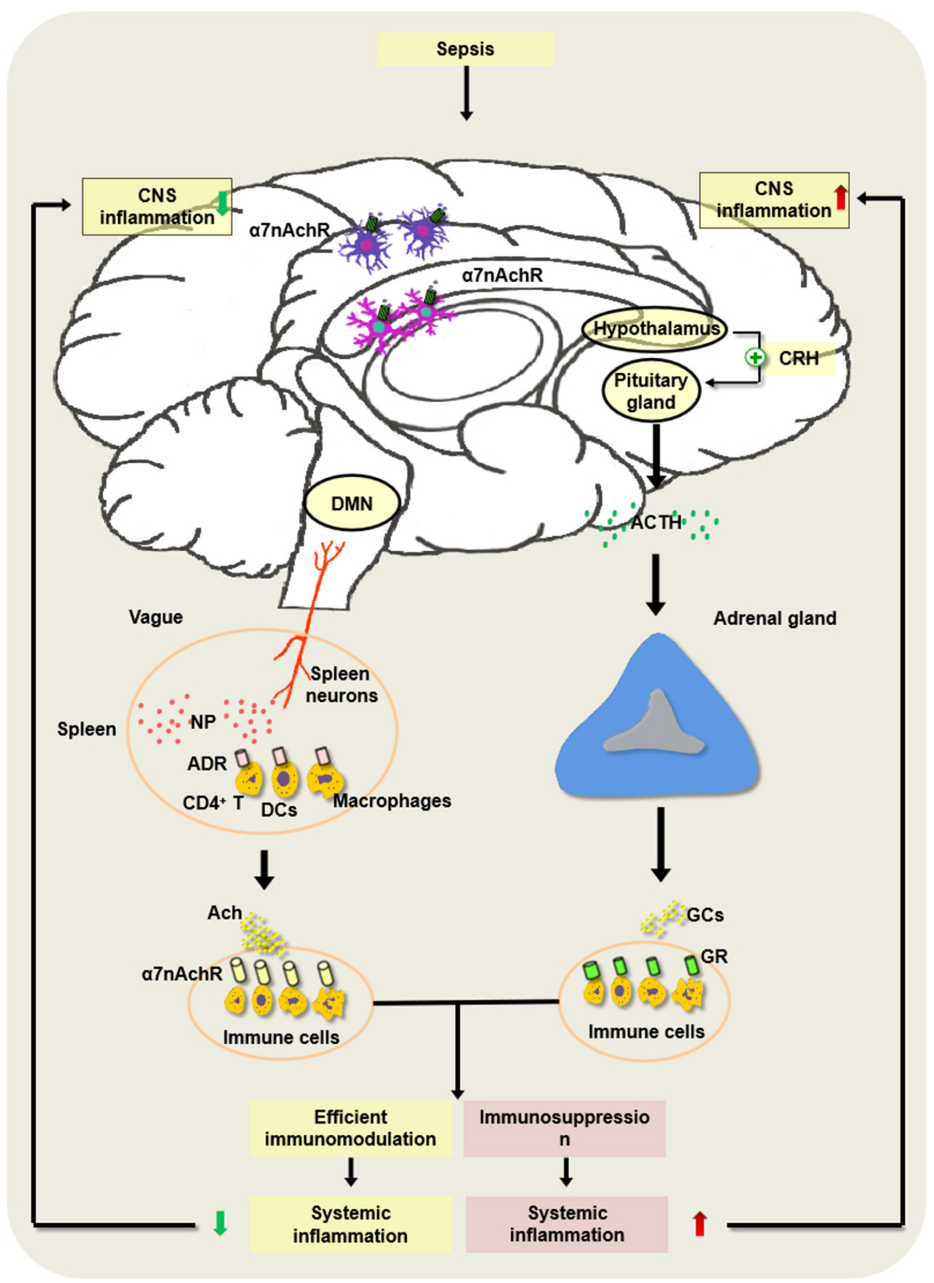
Regulatory mechanism underlying hypothalamic–pituitary–adrenal (HPA) axis and cholinergic anti-inflammatory pathway (CAP) in the development of sepsis. In the pathogenesis of sepsis, the HPA axis is activated *via* the vagus nerve or the humoral pathway, and the hypothalamus and pituitary gland release corticotropin-releasing hormone (CRH) as well as adrenocorticotropic hormone (ACTH). Then, it can augment the production of glucocorticoids (GCs) released by adrenal cortical cells and inhibit excessive inflammation, which may alleviate the sepsis-induced CNS inflammation. However, severe sepsis can result in damage to the HPA axis and the abnormal induction of GC, thereby contributing to immunosuppression and intractable inflammation of the CNS. Of note, the constant brain damage aggravates the abnormality of the HPA axis and forms a vicious cycle under septic exposure. In addition, the activated splenic nerve can release norepinephrine (NP), which binds to the adrenaline receptor (ADR) on immune cells such as CD4^+^ T cells, dendritic cells (DCs), and macrophages to produce ACh. ACh binds to α7 nAChR on the inflammatory cells and suppresses the inflammatory response in the spleen. Nevertheless, the persistent activation of the vagus nerve may lead to the development of host immune suppression following septic insults.

Basically, the HPA axis is a classical anti-inflammatory pathway of the nervous system. Upon septic exposure, the CNS regulates the peripheral inflammatory immune response mainly through the HPA axis. Endotoxin or inflammatory mediators activate the HPA axis through the vagus nerve or humoral pathway, and the hypothalamus releases corticotropin-releasing hormone (CRH) and adrenocorticotropic hormone (ACTH), which will promote the production of glucocorticoid (GC) released by adrenal cortical cells and avoid damage and dysfunction of organs induced by excessive inflammation (101–103). The regulation of the endocrine system based on the HPA axis is relatively slower and longer (Figure 2).

The damage to the HPA axis and the abnormal production of adrenocortical GC appear to be associated with the occurrence and development of sepsis. First, the change in the adrenal microenvironment is an important factor affecting the prognosis of sepsis. An integrated adrenal stress response is critical for the survival of patients with septic shock. Under physiological states, the cortical cells, medulla cells, and epithelial cells in the adrenal gland interact with inflammatory immune cells to form a complex adrenal microenvironment, which plays a key regulatory role in the synthesis and secretion of corticosteroids in sepsis and septic shock (104, 105). A variety of immune cells, including lymphocytes, neutrophils, and macrophages, are associated with adrenal insufficiency and the dysfunction of the HPA axis in the setting of sepsis (106). Under the stimulation of LPS and pathogenic microorganisms, a large number of neutrophils are recruited into the infected sites in adrenal glands by adhesion molecules and chemokines, which may alter the subset of adrenal tissues, leading to dysfunction of the HPA axis and abnormal secretion of adrenal hormones. A study by Carla et al. (107) observed a significant increase in leukocyte infiltration in adrenal glands during sepsis, which was associated with the high mortality of septic animals.

The surface receptors on the adrenal cortical cells and immune cells, such as scavenger receptors, TLR2, and TLR4, are related to the dysfunction of the adrenal glands secondary to sepsis. Kanczkowski et al. found systemic but not adrenocortical-specific knockout of myeloid differentiation protein 88 (MyD88), a TLR adaptor protein, reduced adrenal inflammatory response and HPA axis activation in LPS-induced sepsis (108). A recent study revealed that type B type I scavenger receptors were highly expressed on adrenal cortical cells, which promoted cortisol synthesis by capturing esterified cholesterol; loss of the type I scavenger receptor gene led to adrenal cortical dysfunction, thereby increasing sepsis-induced mortality in mice (109).

GCs produced by the HPA axis are of great value to maintain homeostasis and protect the host from the life threat caused by sepsis (110). The dissonance of the adrenal gland might increase the mortality of patients in septic shock and septic animals because of the disorder in GC hormone metabolism (110, 111). The activated HPA axis enhances the release of GCs from the adrenal gland, which binds to the cytosolic GC receptor (GR) on the cell membrane and participates in many cellular and physiological contexts including interferon (IFN)-γ production, inflammation, and LPS-induced mortality by regulating a series of gene networks. Severe sepsis *per se* can induce the disorder of the corticosteroid system, which results in intractable immunosuppression together with excessive inflammation.

GR is distributed in various kinds of immune cells, e.g., DCs, myeloid cells, macrophages, T cells, and natural killer cells (NKs). It protected the mice from shock induced by LPS and CLP through the upregulation of anti-inflammatory genes, such as glucocorticoid-induced leucine zipper (GILZ), MAPK phosphatase-1 (MKP-1), and programmed death 1 (PD-1), or the inhibition of the proinflammatory genes, including STAT1, IL-1β, and IL-12, in a dimer-dependent way (111–118). Disorder of the HPA axis by modulation of GR might alter the sensitivity of mice to septic challenge. The abnormality of the GR signaling induced by sepsis deteriorated the pathophysiology by decreasing the lactate clearance and increasing the sensitivity of the mice to lactate-mediated toxicity, while the upregulation of GR inhibited the inflammatory response and improved the survival following endotoxic shock (119, 120). Additionally, GR deletion in T cells was associated with the immunosuppressive state in mice subjected to CLP (121).

Recently, several studies have indicated that GC/GR signaling pathways contribute to promoting the capacity of monocytes/macrophages to kill the various particles and bacteria (122, 123). Injection of low-dose GCs or the upregulation of the GILZ markedly protected animals against CLP-induced sepsis by augmenting bacterial clearance (124–126). Of note, GCs help to clean out apoptotic cells and cell debris, which is crucial to tissue repair. It has been shown that the CNS (cerebral cortex, basal forebrain, midbrain, and brainstem) is critically associated with the regulation of immunity and inflammation; moreover, the hypothalamus and the limbic system play central roles in the regulation of neuroendocrine and autonomic nervous systems (127). The CNS can directly innervate the immune organs through the HPA axis and regulate the peripheral immune system. Likely, the information with regard to the peripheral immune–inflammatory response can be transmitted into the CNS through the nervous and humoral pathways to affect neuronal activity (128, 129). The persistent excitation of the HPA axis has negative impacts on the body, and the insufficient HPA axis function also causes adverse effects. Moderate excitation and timely termination of the neuroendocrine system have an important influence on the course of sepsis. However, the precise role and underlying mechanism of the HPA axis in the central–peripheral immune nets are not well understood and need to be further explored.

### Cholinergic Anti-Inflammatory Pathway

In 2003, Tracey et al. (130) first found that the excitation of the vagus nerve protected the brain from endotoxemia and ischemia–reperfusion injury by inhibiting the release of both early and late cytokines such as TNF-α and high-mobility group box-1 protein (HMGB1). This neural pathway is named the CAP. The cholinergic neural pathways exist between the central and peripheral immune systems, which consist of the vagus, the ACh receptor, the spleen, and the splenic nerves (102, 130).

The vagal parasympathetic fibers start from the vagus dorsal motor nucleus (DMN) and stop at the parasympathetic ganglia of the vagal plexus. The emitted postganglionic fibers are distributed in the thoracic and abdominal organs that control the activity of smooth muscles, cardiac muscles, and glands. Strikingly, the vagus nerve can regulate the generation of proinflammatory cytokines. When the vagal nerves sense the stimulation of inflammatory insults, they transmit inflammatory signals to the center neural system. At the same time, the dorsal nucleus of the vagal nerve delivers anti-inflammatory signals to the endothelial reticulate system by ganglion fibers to enhance the synthesis and release of ACh and inhibit the synthesis of proinflammatory cytokines (131–135).

CAP exerts anti-inflammatory effects dependent on the spleen and the splenic nerves. The spleen is an important secondary lymphoid organ. The splenic nerves originate from supraceliac mesenteric neurons and are distributed to the spleen. It was reported that the activation of the vagus nerve or the splenic nerve significantly inhibited the production of TNF-α released by the red pulp and the marginal zone (132, 133, 136). However, the mechanism by which stimulating the vagus or the splenic nerve produces ACh in the spleen is controversial. A previous study speculated that ACh was released from vagal neurons, and it interacted with α7 nAChR on immune cells and ameliorated the inflammatory lesions (137). However, a series of studies implicate that, as an adrenergic nerve, the splenic nerve does not release ACh directly. The stimulated splenic nerve produces norepinephrine (NP), which combines with the adrenaline receptor (ADR) on the surface of CD4^+^ChAT^+^ T cells to initiate the synthesis of ACh, and ACh acts on the inflammatory cells expressing α7 nAChR in the spleen and suppresses the inflammatory response in the spleen (101). The stimulation of the vagus nerve did not reduce proinflammatory mediators in the plasma of sepsis in nude mice lacking T cells. In addition, β-adrenergic receptors on T cells are the important components of splenic nerve signaling (138, 139). Recent studies showed that DCs and macrophages were positively expressed choline acetyltransferase (ChAT), and their activation promoted the ACh synthesis (137) (Figure 2).

The cholinergic pathway regulates systemic inflammation more rapidly and significantly than the HPA axis. The most prominent feature of the CAP is mediated by α7 nAChR. In the CNS, α7 nAChR is located on the surface of various nerve cells including astrocytes, microglia cells, interneurons, and immature granule cells, which are distributed in the basal ganglia, the hippocampus, and the brain gray matter (140–142). It is believed that α7 nAChR can be involved in many psychiatric and neurological disorders including neuroinflammation, Parkinson's disease, and Alzheimer's disease by regulating neurotransmitter release, synaptic plasticity, and signal transduction (143–145). The functions of α7nAChR in the nervous system are determined by location, time, and context. α7 nAChR in hippocampal interneurons is associated with hippocampus-dependent memory, and its activation on excitatory synapses appears to be involved in synaptic activities. However, α7 nAChR on microglia and astrocytes is responsible for the development of neuroinflammation. After being activated by ACh and choline, α7 nAChR can inhibit the expression of proinflammatory mediators by downregulating the phosphorylation of JAK2/STAT3 and reducing nuclear translocation of NF-κB. Li et al. (146) found that ACh protected neurons from inflammatory and apoptotic effects of activated microglia by α7 nAChR. Similarly, it was reported that α7 nAChR activation on astrocytes had a neuroprotective impact *via* inhibiting IL-6 as well as TNF-α secretion and decreasing apoptosis and toxicity of neurons upon LPS stimulation (147–149).

In the periphery immune system, the α7 nicotinic receptor is almost expressed in all kinds of immune cells. The activation of α7 nAChR is evident to suppress the induction of proinflammatory cytokines secondary to the activation of TLRs including TLR2, TLR3, and TLR9 (150). Similarly, the activation of α7 nAChR on macrophages significantly alleviates the LPS-mediated inflammatory processes by downregulating NF-κB-mediated transcription. Moreover, α7 nAChRs on T cells and antigen-presenting cells (APCs) regulate the differentiation of CD4^+^ T cells. Mashimo et al. (151) reported that activated α7 nAChRs on T cells promoted T-cell differentiation, while the activation of α7nAChRs on APCs suppressed T-cell differentiation by decreasing antigen processing.

With the development of the genome-wide association study, a series of studies confirmed that the *CHRNA7* genes encoded for the α7 nAChRs in the human brain and the variations within *CHRNA7* were associated with the abnormal response of CAP (140, 152). In addition, chaperone proteins such as resistance to inhibitors of cholinesterase-3 (RIC3) Ly6h and NACHO could greatly affect the expression and function of nAChRs (143, 153, 154).

Thus, evidence shows that the CAP can abate both systemic and cerebral inflammations resulting from septic challenges. However, immune dysfunction in sepsis appears to be bipolar, accompanied by the excessive inflammatory response or immune suppression at the early or later stage. The constitutive activation of the vagus nerve may lead to immune impairment and infection in septic survivors (155). In the previous studies, we found that HMGB1 in the CNS could induce immune depression of DCs by triggering the hyperactivation of the CAP in CLP-induced septic mice, and the inhibition of cerebral HMGB1 significantly protected the brain against sepsis-induced injury and improved the immune function of splenic T cells (156–158). These data suggest that the nervous system plays an important role in immune surveillance and inflammation control in the bidirectional communication between the brain and the immune system. The efferent signal of the vagal nerve provides a direct pathway to the neural–immune response, and the CNS can use the CNS–peripheral immune axis through the cholinergic neural pathway to modulate the immune response and control the course of sepsis. Nevertheless, the current mechanistic study of the cholinergic neural pathway in the central–peripheral immune regulation of sepsis is still in its infancy.

## Conclusion

As the center of neuroendocrine immune networks, the CNS plays a pivotal role in maintaining the balance between inflammatory response and immunosuppression. In the development of sepsis, uncontrolled neuroinflammation may induce an over-activated inflammatory response or inactivation of peripheral immune cells in sepsis *via* HPA or CAP. At the same time, the peripheral immune system can be fed back to the center and aggravate the progress of SAE, which may form a vicious cycle and cause the disorder of the host immune system. Therefore, it may be a potential target for treating sepsis by alleviating the disharmonious interaction between the central and peripheral immune systems. Many studies from our group and others have revealed that the inhibition of cerebral HMGB1 may attenuate the sepsis-induced brain injury and improve the T-cell-mediated immunity by CPA. However, it remains a new field to explore the management of sepsis by central–peripheral immune mechanisms, and further studies are needed to elucidate the close crosstalk between the CNS and peripheral immune systems following sepsis and septic shock.

## Author Contributions

Y-xL and YY drafted the manuscript. J-pL, W-jL, and YC drew the illustrations. R-mY revised the manuscript. Y-mY conceived and designed the review. All authors contributed to the article and approved the final manuscript.

## Funding

The present work was supported by grants from the National Natural Science Foundation of China (82130062 and 82172124) and the Key Medical Innovation Program of the Chinese People's Liberation Army (18CXZ026).

## Conflict of Interest

The authors declare that the research was conducted in the absence of any commercial or financial relationships that could be construed as a potential conflict of interest.

## Publisher's Note

All claims expressed in this article are solely those of the authors and do not necessarily represent those of their affiliated organizations, or those of the publisher, the editors and the reviewers. Any product that may be evaluated in this article, or claim that may be made by its manufacturer, is not guaranteed or endorsed by the publisher.
